# Angiogenesis Dysregulation in the Pathogenesis of Systemic Sclerosis

**DOI:** 10.1155/2017/5345673

**Published:** 2017-07-16

**Authors:** Francesco Paolo Cantatore, Nicola Maruotti, Addolorata Corrado, Domenico Ribatti

**Affiliations:** ^1^Rheumatology Clinic, Department of Medical and Surgical Sciences, University of Foggia Medical School, Foggia, Italy; ^2^Department of Basic Medical Sciences, Neurosciences and Sensory Organs, University of Bari Medical School, Bari, Italy; ^3^National Cancer Institute “Giovanni Paolo II”, Bari, Italy

## Abstract

Systemic sclerosis is a chronic autoimmune connective tissue disease characterized by vascular injury and fibrosis and by an impaired angiogenesis which cannot ensure an efficient vascular recovery. Vascular injury is responsible for hypoxia and tissual ischemia which are the primary triggers for angiogenesis and are not able to induce a compensatory angiogenesis. This review article is focused on current knowledge on the mechanisms responsible for angiogenesis dysregulation in systemic sclerosis.

## 1. Introduction

Systemic sclerosis (SSc) is a chronic autoimmune connective tissue disease characterized by multisystem involvement with inflammation, vasculopathy, fibrosis of the skin, and internal organs. Vascular injury is an early event in SSc pathogenesis and may be evident before the onset of fibrosis. It plays a central role in promoting Raynaud's phenomenon, digital ulcers, renal damage, and pulmonary arterial hypertension [1–6].

The interaction between autoimmunity, vascular injury, and fibrosis promotes the progressive tissue damage. Among these factors, autoimmunity and vascular injury characterize the earliest phases of the disease and contribute to the progression of fibrosis [7–9].

Angiogenesis, the formation of newly formed capillaries from preexisting vessels via a well-programmed cascade of events, is dysregulated in SSc and cannot ensure an efficient vascular recovery. Vascular injury induces hypoxia and tissual ischemia which are the primary triggers for angiogenesis, but are not able to induce a compensatory angiogenesis.

## 2. Vascular Involvement in SSc

In early stages of SSc endothelial cell injury is followed by vascular remodeling, which is characterized by capillary enlargement, intimal proliferation, telangiectasia, and accumulation of proteoglycans in the arterioles. Later, during the course of the disease, loss of capillaries and small arterioles induces the formation of avascular areas [10, 11]. In larger size vessels, there is vascular occlusion and thrombosis due to endothelial proliferation, fibrotic intimal proliferation, fibrin deposition, and smooth muscle cell hypertrophy [10–12].

A typical clinical feature of SSc is the Raynaud's phenomenon, which is characterized by persistent vasospasm of digital arteries with alternation of ischemia and reperfusion and an increased expression of junctional adhesion molecules (JAMs) [11]. The increase expression of JAMs promotes neutrophils and platelets attachment to endothelial cells. Neutrophils and platelets, in turn, induce the production of superoxide radicals which are responsible for endothelial cell injury [10].

Thus, in SSc an endothelial cell damage has been observed already in early phases of the disease, which probably together with neural dysfunctions, and other intravascular alterations, is in turn involved in inducing vascular injury [3, 4, 10]. Even if etiopathogenesis of endothelial cell damage is still unclear, autoimmunity seems to be responsible for endothelial production of cytokines and adhesion molecules, leading to apoptosis of the endothelial cell [10, 13–15].

Moreover, an imbalance between vasodilator agents, such as nitric oxide, and vasoconstrictor agents, such as endothelin-1 (ET-1), has been observed in SSc and is involved in altered vascular permeability [15]. In fact, increased ET-1 expression is involved in inflammation, vascular fibrosis, and increased smooth muscle cell proliferation [4]. Moreover, the expression of ET-1 receptor type B, called ETB receptor, is reduced in SSc on endothelial cell surface but overexpressed on smooth muscle cells where it is responsible for fibrosis and vasoconstriction [16, 17].

Cipriani et al. [18] have demonstrated that endothelial cells in SSc, under the synergistic effect of transforming growth factor-*β* (TGF-*β*) and ET-1, may transdifferentiate toward myofibroblasts in a process called endothelial-to-mesenchymal transition (EndoMT) by which endothelial cells change their morphological features and assume myofibroblast-like features. By using skin sections obtained by SSc patients, Manetti et al. [19] have recently observed that EndoMT has a key role in inducing endothelial dysfunction and dermal fibrosis in SSc. Macitentan, an ET-1 receptor antagonist, has demonstrated an inhibitory effect in vitro on EndoMT and on fibroblast activation, suggesting its potential role as new therapeutic strategy against fibrosis in SSc [18, 20].

Transdifferentiation of pericytes to myofibroblasts has also been hypothesized in SSc [21]. By considering that pericytes are cells that reside on the wall of the blood vessels and their primary function is to maintain the vessel integrity, their transdifferentiation to myofibroblasts may contribute to vessel instability.

Vascular damage may induce the onset of avascular areas and/or morphologic changes in vessel wall, such as fibrosis. These alterations play a central role in inducing tissual hypoxia which may induce digital ulcers, gangrene and amputation of the extremities, and dysfunction in several internal organs [22–26].

## 3. Vascular Implications and Fibrosis

Angiogenic cytokines, responsible for vessel formation and stabilization, such as vascular endothelial growth factor (VEGF), TGF-*β*, and platelet derived growth factor (PDGF), are also involved in fibrosis. An impaired cross-talk between endothelial cells and perivascular cells, such as pericytes, may induce an abnormal expression of these angiogenic factors in SSc [27]. This is responsible for subsequent vessel instability and perivascular-myofibroblast phenotypic transdifferentiation, which contribute to fibrosis in the skin and internal organs and to the loss of peripheral vascularization [27].

## 4. The Role of Imbalance between Angiogenic and Antiangiogenic Factors

An imbalance between angiogenic and antiangiogenic factors has been observed in several autoimmune diseases [28–30]. In SSc a dysregulation of some angiogenic factors, such as VEGF, fibroblast growth factor-2 (FGF-2), PDGF, TGF-*β*, monocyte chemoattractant protein-1 (MCP-1), stromal cell-derived factor 1 (SDF-1/CXCL12), interleukin (IL)-8, ET-1, and urokinase type plasminogen activator receptors (uPAR), and some antiangiogenic factors, such as angiostatin, thrombospondin-1 (TSP-1), endostatin, platelet factor 4 (PF4/CXCL4), IL-4, and pentraxin 3 (PTX3), has been described (Table 1) [10, 11, 30–36].

High levels of VEGF have been demonstrated in SSc, in spite of an inadequate angiogenesis [37, 38]. Nevertheless, by considering that previous studies did not distinguish between proangiogenic VEGF(165) and antiangiogenic VEGF(165)b isoforms, originated by alternative splicing in the terminal exon of VEGF pre-RNA, Manetti et al. [39] have observed that a switch from proangiogenic to antiangiogenic VEGF isoforms may be responsible for the inefficient angiogenic response in SSc. Recently, increased production of VEGF(165)b has been found in platelets isolated from SSc patients, suggesting a role for platelets in insufficient angiogenesis [40].

Even if numerous angiogenic factors are overexpressed in SSc, reduced levels of Angiopoietin-1 (Ang-1) have been observed in sera of patients with SSc, whereas Ang-2, an antagonist of Ang-1, was upregulated. Moreover, reduced levels of Kallikreins 9, 11, and 12, three serine proteases with angiogenic activity, have been observed in SSc endothelial cell [41, 42].

An increased production of antiangiogenic factors, such as endostatin and angiostatin, has been observed in SSc. Recent evidence shows that endostatin levels are increased in all phases of the disease while angiostatin levels are significantly elevated in late disease and are correlated to lung disease severity [43].

## 5. Reduced Expression of Receptors for Angiogenic Factors

A possible role in the lack of response to angiogenic factors in SSc, despite their overexpression, has been suggested for the reduced expression of some receptors on cell membrane. In fact, a reduced expression of stromal cell-derived factor 1 (SDF1), an angiogenic factor also known as CXC motif chemokine 12 (CXCL12), and its receptor CXCR4 has been found in later stages of disease skin biopsy samples from SSc patients, while they were upregulated in the skin of patients with early SSc, playing probably a role in the inadequate angiogenic response [44]. Nevertheless, contrasting results for VEGF receptor-1 (VEGFR-1) and VEGFR-2 expression have been described in SSc endothelial cells [45–49]. Moreover, overexpression of VEGFR-3 and chemokine receptors, such as CXCR2 (receptor of IL-8) and CXCR6 (receptor of CXCL6), has been found in endothelial cells and dermal fibroblasts isolated from SSc patients [44, 50, 51]. Recently, Tsou et al. [52] have found that increased expression of angiogenic chemokines, such as growth-regulated protein-*γ* (Gro-*γ*/CXCL3) and granulocyte chemotactic protein 2 (GCP-2/CXCL6) in serum and endothelial cells obtained from SSc patients, was unable to induce angiogenesis.

## 6. Impaired Expression of Angiogenic Transcription Factors

Another hypothesis to explain the lack of response to angiogenic factors in SSc is the impaired expression of angiogenic transcription factors, such as Friend leukemia integration-1 (Fli1) and Fos-related antigen 2 (Fra-2). Fli1 acts as a suppressor of collagen transcription in human skin as demonstrated in vivo. The persistent reduced expression of Fli1 in SSc fibroblast cultures has been correlated to abnormal matrix deposition in scleroderma skin. Low Fli1 levels have been correlated to the detachment of preexisting pericytes, extracellular matrix degradation by endothelial proteinases, enhanced migration, proliferation, and cell survival. On the contrary, Fli1 deficiency plays a role in inhibiting tube formation of endothelial cells, suggesting that Fli1 deficiency is probably a consequence of both proliferative obliterative vasculopathy, characterized by occlusion of arterioles and small arteries, and destructive vasculopathy, characterized by loss of small vessels, which are the typical alterations in SSc vasculopathy [53]. On the other hand, high levels of Fra-2 have been seen in SSc patients, and its overexpression has been correlated to increased profibrotic effects of TGF-*β* and PDGF [54–56].

## 7. JAM-A

The reduced expression of JAM-A on endothelial cells surface decreases FGF-2 induced angiogenesis [57] and has been correlated to an increased cleavage of IL-8 and uPAR, two angiogenic factors which are responsible for endothelial cell proliferation, extracellular matrix degradation, and the adhesion of endothelial cells to the extracellular matrix, by MMPs overexpression in fibroblast and endothelial cell [58–60].

## 8. Genetic Polymorphisms

Genetic polymorphisms may also be involved in SSc pathogenesis. Gene polymorphism of uPAR, called UPAR rs344781, has been associated with increased risk of vascular injury in SSc, while gene polymorphism of MMP-12, named MMP-12 rs2276109, has been correlated with diffuse cutaneous SSc and pulmonary fibrosis [61, 62]. Moreover, an increased expression of histone deacetylases-5, an enzyme involved in the control of genes associated with angiogenesis regulation, has been observed in endothelial cells from SSc patients, suggesting a potential role for epigenetic modification in impaired angiogenesis [63].

## 9. *α*-Klotho

Recently, a role for *α*-klotho, a pleiotropic protein, originally described as an antiaging factor, has been suggested in SSc pathogenesis by acting as a powerful proangiogenic factor. This factor plays important pleotropic effects on endothelial cells, by interacting with VEGFR-2 and transient receptor potential canonical-1 (TRPC-1) cation channel to control cellular homeostasis [64]. Mazzotta et al. [65] have found that *α*-klotho is significantly decreased in the microvasculature in SSc skin and that its administration may efficiently improve dermal microvascular endothelial cells from SSc patients functions in vitro.

## 10. A Link between Vascular and Nervous System

Emerging evidences underline the link between vascular and nervous system. In fact, factors responsible for transmitting axonal guidance cues, such members of class III semaphorin (Sema3) family, play an antiangiogenic role in physiological and pathological vascular development. These factors are involved in reducing cell adhesion by disrupting integrin-mediated adhesive structures, resulting in a filopodial retraction in endothelial cells. Recently, by using dermal microvascular endothelial cell cultures from SSc patients, Mazzotta et al. [66] have suggested that a member of Sema family, named Sema3E, by binding to its receptor Plexin-D1 plays probably a role in the dysregulation of angiogenesis and vascular tone control by inducing neurovascular mechanism alterations which are clinically evident above all in the early stage of the disease. A low expression of neuropilin-1, a receptor for both Sema3s and VEGF-A, has been observed in SSc, suggesting a further additional factor involved in impaired angiogenesis [67].

## 11. The Role of Mesenchymal Stem Cells (MSCs) in the Vascular Alteration during SSc: Therapeutic Implications

In SSc patients, MSCs are characterized by senescence [68]. Nevertheless, MSCs may preserve immunomodulatory ability, which might have potential therapeutic implications in SSc. In fact, Cipriani et al. [68] have found increased levels of IL-6 and TGF-*β* in SSc-MSCs. On one hand, increased levels of IL-6 have been considered as an adaptive mechanism to senescence and are responsible for immunosuppressive effects. On the other hand, increased levels of TGF-*β* may be involved in determining both immunosuppressive effect on lymphocyte proliferation and immunoregulatory effects, via inducing expression of CD69 on T cells surface [68]. Moreover, MSCs may differentiate into endothelial cells [69], suggesting a potential therapeutic role in vascular alteration during SSc.

Different sclerotic conditions, including localized scleroderma, have been effectively treated with autologous fat tissue grafting (AFTG). In patients affected by advanced SSc-related perioral thickening and mouth opening limitation, AFTG of the lips has demonstrated an improvement of mouth opening [70]. The efficacy of this treatment has been correlated to the presence of a stem cell population, called adipose-derived MSCs (ATDMSCs) in the adipose tissue. In fact, ATDMSCs may differentiate into endothelial cells and produce angiogenic factors, suggesting a potential role in promoting angiogenesis [71]. ATDMSCs exert also several immunosuppressive and anti-inflammatory effects by inhibiting both proliferation of T and B cells, and the expression of numerous proinflammatory cytokines [72]. Furthermore, adiponectin expression from adipose tissue is responsible for antifibrotic effects [73].

## 12. Concluding Remarks

SSc in the earliest stages is characterized by morphologic alterations in vessel walls, such as fibrosis and capillary loss. Endothelial cell injury plays a central role in promoting these changes, which are responsible for inducing hypoxia. These events lead to an increased angiogenesis. Nevertheless, in SSc patients angiogenesis is not compensatory. The reason of this inefficient angiogenesis in SSc is still unclear. Nevertheless, an imbalance between angiogenic and antiangiogenic factors and a reduced expression of some receptors or cofactors of angiogenic agents has been suggested.

Even if further studies are needed to explain the role of angiogenesis in the pathogenesis of SSc and to elucidate the mechanism responsible for angiogenesis dysregulation, endothelial cell injury and angiogenesis dysregulation seem to play a central role in the pathogenesis of SSc. This may provide a basis for a rational approach to the development of new therapeutic strategy to ensure efficient angiogenesis.

## Figures and Tables

**Table 1:** Angiogenic and antiangiogenic agents involved in SSc: imbalance between these factors is responsible for impaired angiogenesis.

Angiogenic factors in SSC	
Stimulators	Inhibitors
VEGF(165)	VEGF(165)b
FGF-2	TSP-1
PDGF	PF4/CXCL4
TGF-*β*	PTX3
MCP-1	Endostatin
SDF-1/CXCL12	Angiostatin
IL-8	Ang-2
ET-1	
uPAR	
Ang-1	
Kallikrein 9	
Kallikrein 11	
Kallikrein 12	
Gro-*γ*/CXCL3	
gGCP-2/CXCL6	
Fli1	
Fra-2	
*α*-Klotho	

(VEGF: vascular endothelial growth factor; FGF-2: fibroblast growth facor-2; PDGF: platelet derived growth factor; TGF-*β*: transforming growth factor-*β*; MCP-1: monocyte chemoattractant protein-1; SDF-1: stromal cell-derived factor 1; IL-8: interleukin-8; ET-1: endothelin-1; uPAR: urokinase type plasminogen activator receptors; Ang: Angiopoietin; Gro-*γ*: growth-regulated protein-*γ*; gGCP-2: granulocyte chemotactic protein 2; Fli1: Friend leukemia integration-1; Fra-2: Fos-related antigen 2; TSP-1: thrombospondin-1; PF4: platelet factor 4; PTX3: pentraxin 3).
